# Nutrient Use Efficiency of Southern South America Proteaceae Species. Are there General Patterns in the Proteaceae Family?

**DOI:** 10.3389/fpls.2018.00883

**Published:** 2018-06-27

**Authors:** Mabel Delgado, Susana Valle, Marjorie Reyes-Díaz, Patricio J. Barra, Alejandra Zúñiga-Feest

**Affiliations:** ^1^Laboratorio de Biología Vegetal, Facultad de Ciencias, Instituto de Ciencias Ambientales y Evolutivas, Universidad Austral de Chile, Valdivia, Chile; ^2^Centro de Investigación en Suelos Volcánicos, Universidad Austral de Chile, Valdivia, Chile; ^3^Departamento de Ciencias Químicas y Recursos Naturales, Facultad de Ingeniería y Ciencias, Universidad de La Frontera, Temuco, Chile; ^4^Center of Plant, Soil Interaction and Natural Resources Biotechnology, Scientific and Technological Bioresource Nucleus (BIOREN), Universidad de La Frontera, Temuco, Chile; ^5^Facultad de Ciencias Agrarias, Instituto de Ingeniería Agraria y Suelos, Valdivia, Chile

**Keywords:** phosphorus and nitrogen use efficiency, photosynthesis rate, specific leaf area, cluster roots, chilean soils

## Abstract

Plants from the Proteaceae family can thrive in old, impoverished soil with extremely low phosphorus (P) content, such as those typically found in South Western Australia (SWA) and South Africa. The South Western (SW) Australian Proteaceae species have developed strategies to deal with P scarcity, such as the high capacity to re-mobilize P from senescent to young leaves and the efficient use of P for carbon fixation. In Southern South America, six Proteaceae species grow in younger soils than those of SWA, with a wide variety of climatic and edaphic conditions. However, strategies in the nutrient use efficiency of Southern South (SS) American Proteaceae species growing in their natural ecosystems remain widely unknown. The aim of this study was to evaluate nutrient resorption efficiency and the photosynthetic nutrients use efficiency by SS American Proteaceae species, naturally growing in different sites along a very extensive latitudinal gradient. Mature and senescent leaves of the six SS American Proteaceae species (*Embothrium coccineum, Gevuina avellana, Orites myrtoidea Lomatia hirsuta, L. ferruginea*, and *L. dentata*), as well as, soil samples were collected in nine sites from southern Chile and were subjected to chemical analyses. Nutrient resorption (P and nitrogen) efficiency in leaves was estimated in all species inhabiting the nine sites evaluated, whereas, the photosynthetic P use efficiency (PPUE) and photosynthetic nitrogen (N) use efficiency (PNUE) per leaf unit were determined in two sites with contrasting nutrient availability. Our study exhibit for the first time a data set related to nutrient use efficiency in the leaves of the six SS American Proteaceae, revealing that for all species and sites, P and N resorption efficiencies were on average 47.7 and 50.6%, respectively. No correlation was found between leaf nutrient (P and N) resorption efficiency and soil attributes. Further, different responses in PPUE and PNUE were found among species and, contrary to our expectations, a higher nutrient use efficiency in the nutrient poorest soil was not found. We conclude that SS American Proteaceae species did not show a general pattern in the nutrient use efficiency among them neither with others Proteaceae species reported in the literature.

## Introduction

The flowering plant family Proteaceae is mostly constituted by non-mycorrhizal species that form specialized “proteoid” or cluster roots, which efficiently mobilize nutrients from the soil by releasing organic compounds (Lamont, 2003; Shane and Lambers, 2005; Lambers et al., 2006, 2015b). Naturally, Proteaceae species can be found in the Southern Hemisphere, where South Western (SW) Australian Proteaceae species are by far the most abundant, followed by South African Proteaceae species (Pate et al., 2001). In those regions, Proteaceae species inhabit some of the most phosphorus (P)-impoverished soils in the world (Shane and Lambers, 2005). Several studies have shown that the success of these plants relies on specific adaptations to thrive on severely P-impoverished soils. Lambers et al. (2015a) summarized very well several traits that SW Australian Proteaceae possess, highlighting: (i) “Efficient P resorption from senescing to mature leaves” and (ii) “Exceptionally high photosynthetic P use efficiency (PPUE).” The high levels of P resorption in leaves have been associated with up-regulation of extra and intracellular acid phosphatases and RNase activities in senescing leaves (Shane et al., 2014). Meanwhile, the high PPUE in these plants have been linked with differentiated P allocation patterns, such as extensive replacement of phospholipids by galactolipids and sulfolipids during leaf development (Lambers et al., 2012b) and with very low P allocation to rRNA in young leaves (Sulpice et al., 2014). In addition, a recent study by Hayes et al. (2018) compared leaf cell-specific nutrient concentrations and distributions for a phylogenetically disperse range of Proteaceae species from the extremely P-impoverished soils of SWA and the relatively P-rich soils of South America: Brazil and Chile. These authors assert that only those species from extremely P-impoverished habitats have higher PPUE by preferentially allocate P to photosynthetic mesophyll cells rather than epidermal cells, which suggests some functional divergence among Proteaceae species.

In Southern South America (SSA), where the soils are younger and nutrient richer than in SWA (Lambers et al., 2012a), only six members of the Proteaceae family can be naturally found. Three Southern South (SS) American species, *Embothrium coccineum* J.R. Forst. & G. Forst*., Lomatia hirsuta* Lam., and *Orites myrtoidea* Poepp. & Endl., are shade-intolerant and colonizers of young disturbed soils. Whereas, the other members, *L. ferruginea* Cav. R. Br*., L. dentata* Ruiz et Pavon R.Br., and *Gevuina avellana* Mol., are semi shade-tolerant, requiring protection from direct sunlight in the early stages of their life (Donoso, 2006). The geographical distribution varies among these species, being possible to find a large range of climatic and edaphic conditions in the sites where they inhabit. On the one hand, *L. hirsuta, L. dentata*, and *G. avellana* have their optimum distribution in the warm and deciduous forest biotopes of *Nothofagus obliqua* (35–44° S). While, *E. coccineum* and *L. ferruginea* have a wider distribution (35–56° S) with high presence in the cold biotopes of the Patagonian rainforests (Steubing et al., 1983). On the other hand, the endemic shrub *O. myrtoidea* is the Proteaceae species with the narrowest distribution, growing only in the Andes Mountains (35–38° S) (Hechenleitner et al., 2005). In general, there are few studies about SS American Proteaceae species and most of them have been focused on factors controlling cluster root formation (Donoso-Ñanculao et al., 2010; Zúñiga-Feest et al., 2010; Delgado et al., 2013; Piper et al., 2013). Thus, there are scarce and contradictory information about leaf traits of SS American Proteaceae and how this affects the cycling of nutrients, such as P and nitrogen (N), in their natural ecosystems. Lambers et al. (2012a), proposed that *E. coccineum* growing in P-rich volcanic soils can act as “ecosystem engineers,” because maintain high P levels in senescent leaves, as a result of its low P resorption. Therefore, through the leaf drop provides P in the leaf litter to neighboring plants. However, this hypothesis has recently been refuted by Fajardo and Piper (2015), who found that *E. coccineum* growing in Chilean Patagonia have high foliar P and N resorption, and their shed litter is not richer in nutrient compared to that of other co-occurring species. These contrasting approaches are probably because the nutrient resorption is highly variable depending on the species and nutrient availability in the soil. In this context, Hayes et al. (2014) found that P concentration in leaves of several species (including Proteaceae) declined from youngest to oldest (P-poor) soils, while P resorption efficiency increases from 0 to 79%. The variation in leaf N concentration with soil age was less evident. However, leaf N-resorption efficiency was greatest on the youngest, N-poor soils. These authors also found that SW Australian Proteaceae shows higher leaf P resorption than other plant families inhabiting the same soil.

Although the evidence indicates that SW Australian Proteaceae species are efficient in the use of nutrients, there is no available information about the nutrient use efficiency of the SS American Proteaceae species naturally adapted to grow in a wide range of climatic and edaphic conditions. Therefore, we explored the nutrient (P and N) resorption efficiency and the photosynthetic use efficiency of P (PPUE) and N (PNUE) in leaves of six SS American Proteaceae species growing in a large geographical range (≈ 1,800 km). We also evaluated the leaf N:P ratio to determine which is the limiting nutrient for SS American Proteaceae species. We hypothesized that species growing in nutrient-poor soils have higher resorption efficiency and photosynthetic nutrient use efficiency (of P and N) in their leaves than species growing in nutrient-rich soils. The aim of this study was to evaluate leaf traits related to nutrient use efficiency of Proteaceae species naturally growing in different sites along a large latitudinal gradient (14°), and to compare our results with the literature on Proteaceae species from severely P-impoverished soils (e.g., SWA and South Africa).

## Materials and methods

### Collection sites and leaf sampling

In Chile, there is a broad range of climatic and edaphic conditions where Proteaceae species grow naturally (Tables 1, 2). We selected nine sites from different geographical locations during spring of 2015 and 2016 (Table 1). The weather conditions of the selected sites vary from cold (such as in Antuco, Aysén and Torres del Paine sites) to more temperate zones (all other evaluated sites), and from superhumid regions (e.g., Aysen) to regions with lower precipitation (e.g., Torres del Paine; Table 1). The soils where these species grow vary widely in origin and nutrient availability (Table 2). Most of the selected soils are of volcanic origin with different stages of development; from young rocky soils (e.g., Antuco, Ensenada) to more developed soils with well-defined horizons (e.g., Anticura, Cochamó, Aysen). Besides, we selected soils from different origins such as metamorphic rocks (Nahuelbuta; CIREN, 1999), fluvioglacial terraces (Chaiguata; CIREN, 2003), and glacial sediments (Torres del Paine; Díaz-Vial et al., 1960).

**Table 1:** Geographical parameters from different studied populations where Chilean Proteaceae grow naturally.

**Populations**	**Coordinates**	**Annual rainfall (mm)**	**Temperature (°C)**			**Elevation (m asl)**	**Edaphic Zone**	Soil type^*^
			**Annual**	**Min**	**Max**			
Antuco	37° 23' S−71° 25'W	2012	6.8	1.1	14.0	1050	Wet mediterranean	Typic haploxerand
Nahuelbuta	37° 42' S−73° 13' W	1530	12.3	7.6	17.7	346	Wet mediterranean	Rhodic palehumult
Anticura	40° 3' S−72° 1' W	1641	13.9	9.2	18.7	350	Wet mediterranean	Acrudoxic Hapludand
Ensenada	41° 09' S−72°34' W	2021	10.7	7.3	14.6	39	Wet mediterranean	Andean recent terraces
Cochamó	41° 52' S−72° 28' W	1982	11.0	7.6	15.2	40	Wet mediterranean	Acrudoxic hapludand
Cucao	42° 07' S−73° 56' W	1942	10.4	6.9	14.4	14	Wet mediterranean	Typic hapludand
Tantauco	42° 37' S−74° 5.5' W	1942	10.4	6.9	14.4	150	Wet mediterranean	Fluvioglacial terraces
Aysén	45° 27' S−72° 44' W	2941	9.0	4.5	14.1	8	Rainy	Oxyaquic fulvudand
Torres del Paine	51° 22' S−72° 50' W	722	7.4	1.5	12.6	118	Magellan	Glacial sediments

The climatic variables were collected from the nearest Chilean weather stations reported by Luebert and Pliscoff (2006). Edaphic zones of Chile were described by Casanova et al. (2013).

* *Whenever the classification of the soil taxonomy system (USDA) was missing, the soil type was described according to the origin of its material*.

**Table 2:** Chemical analyses of soil collected in the natural habitats of Chilean Proteaceae.

**Latitude S**	**37.23°**	**37.42°**	**40.30°**	**41.09°**	**41.52°**	**42.07°**	**42.37°**	**45.27°**	**51.22°**
	**(*n* = 15)**	**(*n* = 6)**	**(*n* = 8)**	**(*n* = 3)**	**(*n* = 15)**	**(*n* = 3)**	**(*n* = 3)**	**(*n* = 3)**	**(*n* = 5)**
Mineral N (mg kg^−1^)	25.5 (1.9)	26.8 (1.2)	19.6 (1.5)	24.5 (2.1)	14.35 (0.5)	47.8 (8.7)	36.1 (1.1)	25.9 (1.4)	24.2 (1.2)
P-Olsen (mg kg^−1^)	17.3 (2.5)	2.0 (0.2)	12.4 (5.1)	2.5 (0.3)	2.56 (0.3)	6.8 (2.0)	3.65 (1.0)	11.4 (0.9)	2.92 (0.9)
pH (H_2_O)	6.1 (0.1)	5.3 (0.1)	5.9 (0.2)	6.4 (0.1)	6.12 (0.0)	5.1 (0.2)	4.43 (0.0)	5.5 (0.0)	5.86 (0.1)
pH (CaCl_2_)	5.3 (0.1)	4.3 (0.1)	4.9 (0.1)	5.6 (0.0)	5.15 (0.1)	4.0 (0.3)	3.32 (0.1)	4.8 (0.1)	5.09 (0.1)
Organic C (g 100 g^−1^)	6.2 (0.8)	4.5 (0.5)	7.52 (0.0)	0.04 (0.0)	6.30 (0.5)	16.0 (3.6)	5.24 (0.0)	19.2 (0.0)	2.89 (0.8)
Al (cmol_c_ kg^−1^)	0.1 (0.0)	1.9 (0.2)	1.9 (0.5)	0.02 (0.0)	2.90 (0.9)	2.1 (1.1)	3.53 (0.5)	2.8 (0.6)	0.36 (0.2)
K (cmol_c_ kg^−1^)	0.5 (0.1)	0.2 (0.0)	1.2 (0.1)	0.004 (0.0)	0.94 (0.0)	0.8 (0.2)	0.17 (0.0)	1.5 (0.0)	0.14 (0.0)
Na (cmol_c_ kg^−1^)	0.1 (0.0)	0.01 (0.0)	1.2 (0.0)	0.01 (0.0)	1.16 (0.0)	1.1 (0.1)	0.14 (0.0)	1.4 (0.0)	0.03 (0.0)
Ca (cmol_c_ kg^−1^)	9.9 (2.1)	0.6 (0.2)	9.0 (1.7)	0.10 (0.0)	5.63 (0.6)	3.2 (1.1)	0.28 (0.1)	12.9 (0.3)	1.85 (0.7)
Mg (cmol_c_ kg^−1^)	2.0 (0.4)	0.3 (0.0)	3.0 (0.2)	0.01 (0.0)	2.71 (0.1)	3.7 (0.9)	0.62 (0.1)	4.6 (0.0)	0.25 (0.1)
Al saturation (%)	5.6 (2.9)	63.6 (6.1)	12.2 (3.3)	14.3 (8.2)	22.63 (6.7)	18.3 (7.3)	74.2 (3.8)	11.9 (2.3)	18.66 (6.0)
ECEC (cmol_c_ kg^−1^)	12.5 (2.6)	3.0 (0.2)	16.3 (2.1)	0.13 (0.0)	12.71 (1.4)	10.9 (1.7)	4.74 (0.5)	22.3 (1.1)	2.62 (0.9)
Total N (g kg^−1^)	4.1 (0.8)	1.9 (0.2)	2.9 (0.6)	0.04 (0.0)	4.1 (0.3)	9.8 (1.2)	2.84 (0.0)	10.7 (0.1)	1.82 (0.5)
Total P (mg kg^−1^)	520 (48)	233.8 (23)	285.6 (35)	81.6 (7.2)	302.2 (19)	622 (172)	63.1 (8.5)	951.6 (45.8)	384.1 (98)

Each value corresponds to a mean of samples ± standard error in brackets.

*ECEC, effective cation-exchange capacity*.

In order to evaluate leaf traits related to nutrient use efficiency on Proteaceae species naturally growing in different soils, we selected randomly 4–10 individuals of each species (*Embothrium coccineum, Gevuina avellana, Orites myrtoidea Lomatia hirsuta, L. ferruginea*, and *L. dentata*) present at each sampling site and collected leaf samples. The height of the trees ranged from 1.5 to 3.0 m and the branches holding the collected leaves were located at a maximum of 2 m above ground. The leaves were classified in mature: green leaves and without visible signs of damage, and senescent leaves: yellow-brown leaves still attached to the branch. The leaves collected were taken to the laboratory for further analysis.

### Soil collection and chemical analyses

A minimum of three soil samples were collected at 0–20 cm depth and taken to the laboratory for further chemical analysis (Table 2). Soil samples were sieved (2 mm) to remove organic material (e.g., roots, litter) and other large debris. Subsequently, homogenized soil samples were air-dried and chemical analyses were performed. Briefly, plant-available soil P (Olsen) was measured by extracting soil with 0.5 M NaHCO_3_ at pH 8.5 (Olsen and Sommers, 1982) and determined colorimetrically applying the phospho-antimonyl-molybdenum blue complex method (Drummond and Maher, 1995). The soil's mineral N was determined after Kjeldalh acid-digestion. Organic matter was determined following the wet digestion method by Walkley and Black (1934). Total P was measured after soil ignition (550°C, 1 h) and subsequent digestion in nitric acid and perchloric acid (1:1), to spectrophotometrically determine the phospho-antimonyl-molybdenum blue complex. Soil pH was determined in a 1:2.5 soil to solution ratio in both, water and CaCl_2_ (0.01 M). Exchangeable cations [potassium (K), sodium (Na), calcium (Ca), and magnesium (Mg)] and exchangeable aluminum (Al) were determined according to Sadzawka et al. (2004a) by extraction in ammonium acetate (1M, pH 7.0) and KCl (1 M) solutions, respectively, and measured by atomic absorption and emission spectrophotometer, AAES. Effective cation-exchange capacity (ECEC) was calculated by the sum of the cations.

### Chemical analysis in leaves

The mature and senescent leaves were washed with distilled water and later dried at 60°C for 72 h. The dried samples were ground to a powder and used to analyze P and N concentrations. Phosphorus concentrations were determined colorimetrically using the vanado-phosphomolybdate method, once the grinded sample was calcined and digested in acid. Nitrogen concentrations, were determined by Kjeldahl distillation after acidic digestion (Sadzawka et al., 2004b). The N:P ratio of concentrations in leaves was used as an indicator of N or P limitation, where values below 10 are indicative of N limitation, values over 16 indicate P limitation, and values of between 10 and 16 indicate that plant growth is equally limited by N and P (Koerselman and Meuleman, 1996; Güsewell, 2004).

### Photosynthetic nutrient (P and N) use efficiency

In order to evaluate and compare the photosynthetic nutrient use efficiency per leaf unit, of phosphorus (PPUE) and nitrogen (PNUE), two sites, Ensenada (41.2° S) and Cochamó (41.5° S) were selected. We chose these sites because of the closeness between them, similar climatic conditions and flora composition and, especially, for their contrasting concentrations of total P and N in the soil. The PPUE and PNUE were calculated according to Hidaka and Kitayama (2009) by combining data on photosynthesis per unit leaf area divided by leaf P or N concentration, as shown in the following equation:

$$\begin{array}{l} \text{PPUE or PNUE }\left(\text{μmol }{\text{CO}}_{\text{2}}\;{\text{g}}^{-1}\text{P or N }{\text{s}}^{-\text{1}}\right)\\ \;=\frac{\left(A\left(\text{μmol }{\text{m}}^{2}\;{\text{s}}^{-1}\right)\times \text{SLA}({\text{cm}}^{\text{2}}\;{\text{g}}^{-1}\right)/10,000}{\left[\text{P}\right]\;\text{or}\left[\text{N}\right]} \end{array}$$

Where A corresponds to measurements of photosynthesis rates, SLA to specific leaf area and [P] or [N] to P or N concentration.

The measurements of A were made in fully expanded green leaves (10 measurements for each plant, *n* = 4) using an infrared gas analyzer (ADC IRGA-LCA4—Analytical Development Co. Hoddesdon, UK) between 10:00 and 12:00 a.m. of a clear, sunny spring day. These determinations were performed in ambient CO_2_ and temperature but the photosynthetic photon flux density (PPFD) was set to 400 μmol photons m^−2^ s^−1^. We decided to use this PPFD because according to previous determinations of light saturation curves for photosynthesis all SS American Proteaceae species reach their maximum photosynthetic response at that value (Zúñiga-Feest, A. Unpublished data; Castro-Arevalo et al., 2008).

To determine SLA values, the area of some mature leaves were measured with an area folio-meter (LI-COR Model LI-3100 Area Meter). The leaves were then dried at 60°C for 72 h to determine the leaf dry mass (DM). SLA was calculated dividing the area of the leaf by its respective DM. Chemical analysis of N and P in leaves were performed as previously described.

### Leaf phosphorus and nitrogen resorption efficiency

The percentage of P and N in leaves, which has been exported before dying, defines the leaf resorption efficiency of P and N, and was calculated according to Shane et al. (2014) by the difference between the P or N concentration of mature and senescent leaves, divided by the P or N of the mature leaves.

### Statistical analysis

One-way ANOVAs were applied separately with *post-hoc* Tukey tests to determine significant differences (*P*-value ≤ 0.05) in P and N concentrations in leaves, among species within the same site and among sites for the same species. The same test was applied to determine significant differences in P and N resorption efficiency, PNUE, PPUE, SLA, and P and N concentration per unit of leaf area. All data passed the normality and equal variance tests after natural logarithm transformations. Correlations between leaf traits and edapho-climatic conditions were tested by linear regression. A principal component analysis (PCA) was performed to associate the edapho-climatic variables of the different locations with the plant variables. Only the variables having loads higher than 0.5 (loads > 0.5, Rodrigues de Lima et al., 2008) were considered as explanatory variables of the total variation among sites. ANOVAs analyses were performed using the Statistica 7.0 software, whereas the PCA was conducted in R Studio program.

## Results

### Collection sites and species

**Figure 1:**
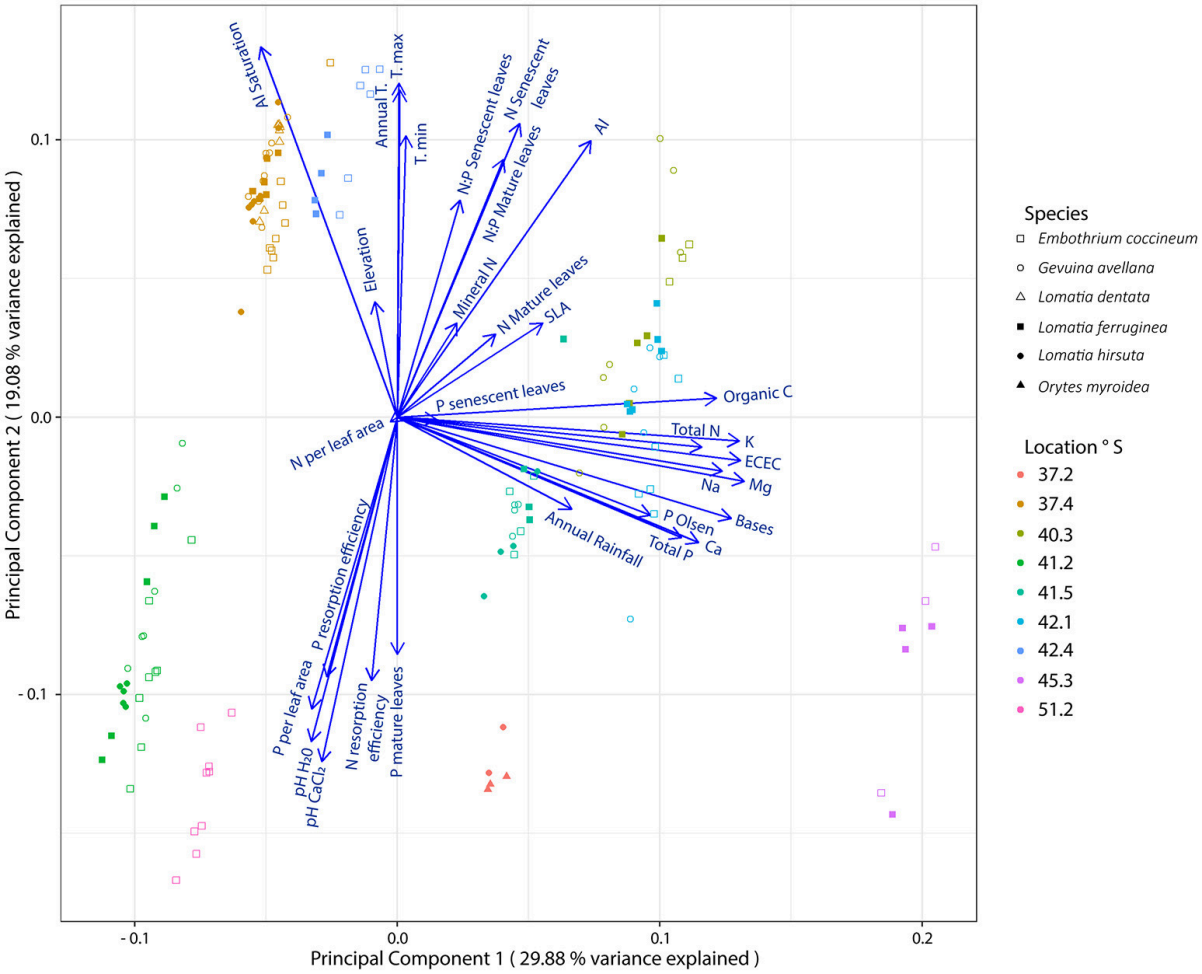
Principal component analysis representing plant measurements from leaves of six Southern South American Proteaceae species and edapho-climatic variables of nine sites where these species grow naturally. Plant measurements: Phosphorus (P) in mature and senescent leaves (mg g^−1^), Nitrogen (N) in mature and senescent leaves (mg g^−1^), P and N resorption efficiency (%), N:P mature leaves, N:P senescent leaves, specific leaf area (SLA; cm^2^ g^−1^), P and N per leaf area (μg cm^2^). Soil variables: mineral N (mg kg^−1^), P Olsen (mg Kg^−1^), pH (H_2_O), pH (CaCl_2_), Organic Carbon (Organic C; g 100 g^−1^), Aluminum (Al; cmol_c_ kg^−1^), Potassium (K; cmol_c_ kg^−1^), Sodium (Na; cmol_c_ kg^−1^), Calcium (Ca; cmol_c_ kg^−1^), Magnesium (Mg; cmol_c_ kg^−1^) interchangeables, Sum of Bases (bases), Effective Cation-Exchange Capacity (ECEC), Al Saturation (%), total N (mg kg^−1^) and total P (mg kg^−1^). Climatic variables: annual rainfall (mm), annual temperature (Annual T., °C), minimal temperature (T. min; °C), maximal temperature (T. max; °C) and Elevation (meters above sea level; m asl).

From analyses performed on edapho-climatic and plant variables, we found that the groups of the species in the PCA were joined according to similar soil chemical characteristics and/or weather conditions of the sites where they grow (Figure 1). Thus, plants from Ensenada (41.2° S) and Torres del Paine (51.2° S) were grouped, likely since both are rocky sites with coarse soil material (mainly coarse sand) containing very low soil organic C content (Table 2). In contrast, plants growing in Anticura (40.3° S), Cochamó (41.5° S) and Cucao (42.1° S) were grouped together likely due to their similar weather conditions, organic C, sum bases and total P and N in the soil. The species from the sites of highest soil Al saturation content and low soil P availability (P-Olsen), Tantauco (42.4° S) and Nahuelbuta (37.4° S), were grouped in the upper quadrant of the left panel. Whereas, the species from Antuco (37.2° S), the site with the lowest Al saturation and highest soil P availability (P-Olsen), were located on the opposite side. Finally, plants from Aysén (45.3° S) were grouped alone because this site present the highest content of N and P in the soil. The PCA also revealed significant correlations (*P* ≤ 0.05) between some plant variables with edapho-climatic conditions. Therefore, P resorption efficiency was correlated with maximum temperature (*R*^2^ = −0.42) and soil pH (pH-H_2_O, *R*^2^ = 0.42; pH-ClCa_2_, *R*^2^ = 0.44). The N resorption efficiency was correlated with Al saturation (*R*^2^ = −0.46) and soil pH (pH-H_2_O, *R*^2^ = 0.46; pH-ClCa_2_, *R*^2^ = 0.40). Meanwhile, the N:P ratio in mature leaves was positively correlated with annual (*R*^2^ = 0.60) and minimum (*R*^2^ = 0.48) temperature and negatively correlated with maximum temperature (*R*^2^ = −0.62). Similar tendency was observed for N:P ratio in senescent leaves.

### Leaf phosphorus and nitrogen concentrations and their resorption efficiency

The nutrient (P and N) concentrations and percentages of nutrient resorption differ among species and over their geographic and edaphic distributions. In general, *E. coccineum* showed the highest values of P and N concentrations in mature leaves compared to the other Proteaceae species evaluated. Thus, the concentrations of P and N were significantly higher in two and four of the nine sites evaluated, respectively (Supplementary Table S2). Thereby, P concentrations in *E. coccineum* ranged from 0.56 to 0.99 mg P g^−1^ and were followed by those for *L. ferruginea* (0.40–0.88 mg P g^−1^), *G. avellana* (0.36–0.67 mg P g^−1^), *L. hirsuta* (0.35–0.63 mg P g^−1^), *L. dentata* (0.35 mg P g^−1^), and *O. myrtoidea* (0.35 mg g^−1^). The same tendency was found in N concentrations in mature leaves, where values for *E. coccineum* ranged from 10.4 to 18.9 mg N g^−1^ DW and were followed by *L. ferruginea* (6.89–13.38 mg N g^−1^), *G. avellana* (8.19–12.1 mg N g^−1^), *L. hirsuta* (5.99–12.3 mg N g^−1^), *L. dentata* (9.96 mg N g^−1^), and *O. myrtoidea* (7.11 mg N g^−1^). In general, similar tendency was found in senescent leaves, where *E. coccineum* had the highest concentrations of P and N in leaves, whereas *O. myrtoidea* had the lowest (Supplementary Table S2). Correlation between total soil P and P in mature leaves (*R*^2^ = 0.028; *P* = 0.051) and between total soil N and N in matures leaves (*R*^2^ = 0.064; *P* = 0.211) was not found. Likewise, correlation between total soil P and P in senescent leaves (*R*^2^ = 0.173; *P* = 0.038) and between total soil N and N in senescent leaves (*R*^2^ = 0.025; *P* = 0.068) was not found either.

**Figure 2:**
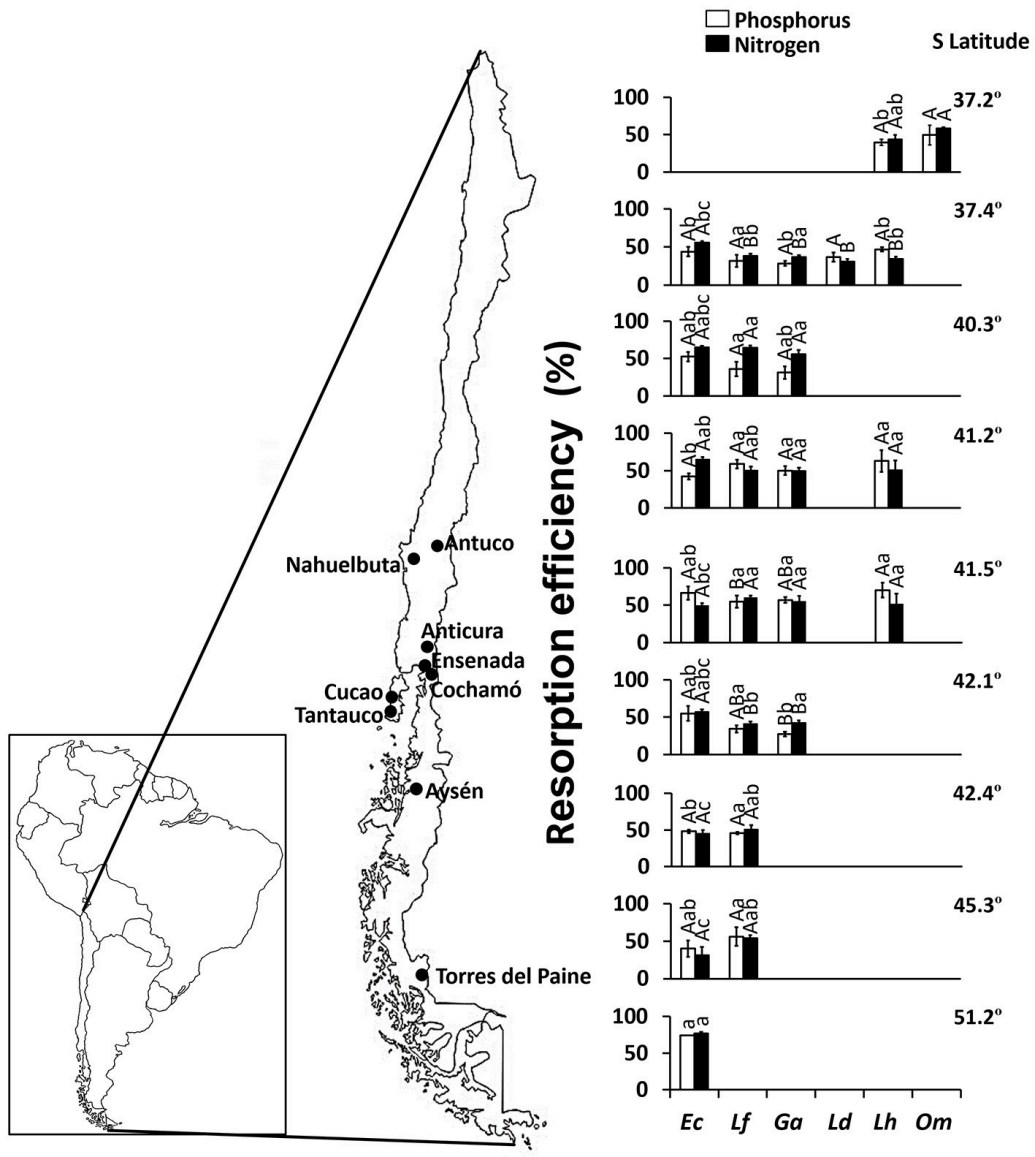
Phosphorus (P) and nitrogen (N) resorption efficiencies in leaves of *Embothrium coccineum* (Ec), *Gevuina avellana* (Ga), *Lomatia ferruginea* (Lf), *Lomatia dentata* (Ld), *Lomatia hirsuta* (Lh), and *Orites myrtoidea* (Om) growing in their natural habitat. Each value corresponds to a mean of 4–10 samples ± standard error. Different capital letters indicate significant differences among species within the same site and different lower-case letters indicate significant differences among sites within the same species (*P* ≤ 0.05).

Combining all species and sites, mean P and N resorption efficiencies were 47.7 and 50.6%, respectively (Figure 2). *Embothrium coccineum* reached the highest values of P and N resorption efficiency (74.5 and 76.9%, respectively), whereas *L. dentata* showed the lowest values (28.5 and 37.0%, respectively). No correlation was found neither between total soil P and P resorption efficiency (*R*^2^ = 0.007; *P* = 0.347) nor between total soil N and N resorption efficiency (R^2^ = 0.025; *P* = 0.074) in leaves.

### Nitrogen: phosphorus ratios for SS american proteaceae

**Figure 3:**
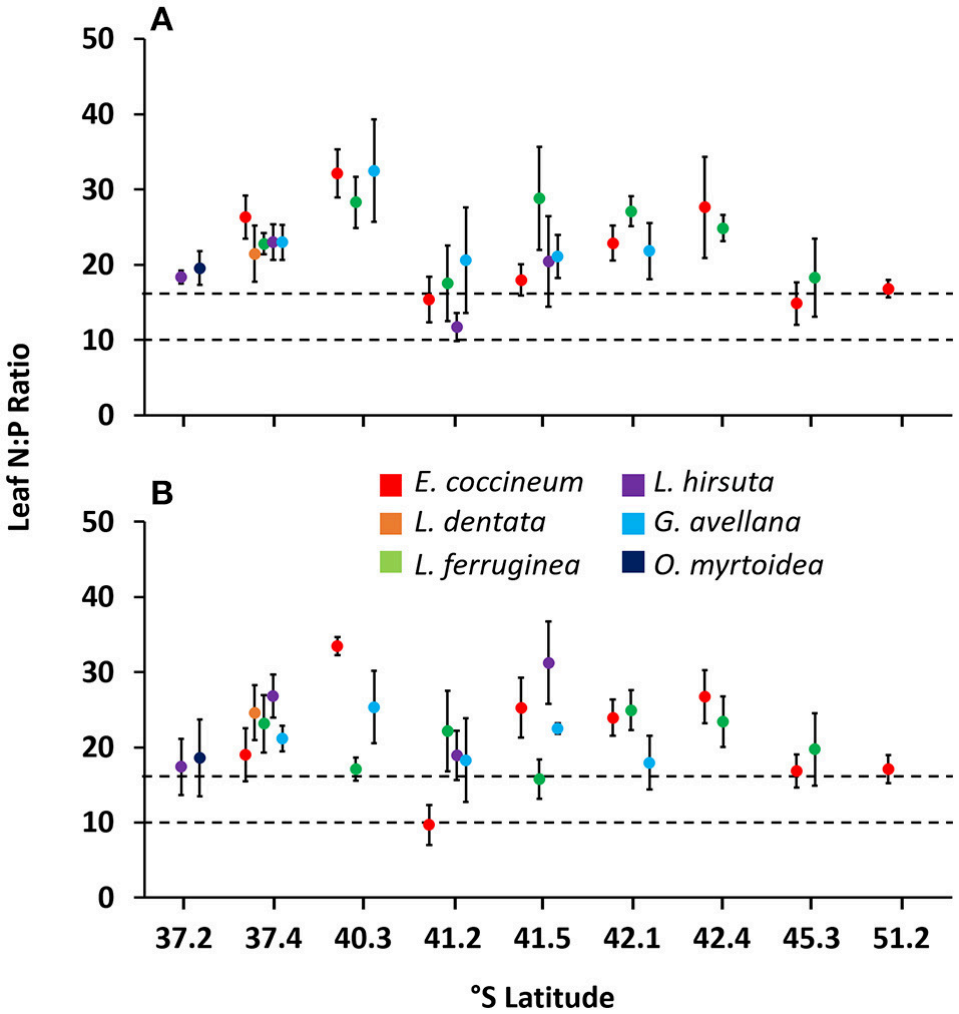
Ratio of N:P concentrations in mature **(A)** and senescent **(B)** leaves of six Southern South American Proteaceae species growing in their natural habitat. Horizontal lines indicate N limitation (values < 10), P limitation (values >16) or both, N and P limitation (values 10–16) in leaves. Each value corresponds to a mean of 4–10 samples ± standard error.

In general, all species studied had on average N:P ratios > 16 in mature and senescent leaves (Figures 3A,B) and, according to the limits of P and N for vegetation proposed by Koerselman and Meuleman (1996), this indicates P limitation in the leaves. Despite this, in some sites with young rocky soil (e.g., 41.2° S, the poorest site evaluated in this study), *L. hirsuta* and *E. coccineum* were co-limited by P and N.

### Photosynthetic nutrient use efficiency per unit leaf nitrogen (PNUE) and phosphorus (PPUE)

In the nutrient richest site (41.5° S), the shade-intolerant species (*E. coccineum* and *L. hirsuta*) had the highest photosynthetic rates compared to the more shade-tolerant species (*G. avellana* and *L. ferruginea*), while in the nutrient poorest site no significant differences among species were found (Table 3). The PNUE and PPUE were significantly affected by the site, species and the interaction between them. Thus, for *E. coccineum*, the highest PNUE and PPUE was found in the nutrient richest site, which was significantly higher compared to the other species evaluated (except *L. ferruginea*, which had similar PPUE at both sites). The lowest PNUE and PPUE was found in *L. ferruginea* in the nutrient poorest site, whereas *G. avellana* and *L. hirsuta* had significantly higher PNUE in the nutrient poorest compared to that of the nutrient richest site, although no significant differences for the PPUE were found for these species (Table 3).

**Table 3:** Rates of photosynthesis, photosynthetic nitrogen use efficiency (PNUE) and photosynthetic phosphorus use efficiency (PPUE) per leaf unit in leaves of *Embothrium coccineum* (Ec), *Gevuina avellana* (Ga), *Lomatia ferruginea* (Lf), and *Lomatia dentata* (Ld) growing in their natural habitat.

**Site**	**Species**	**Photosynthesis rate (μmol CO_2_ m^−2^ s^−1^)**	**PNUE (μmol CO_2_ g^−1^ N s^−1^)**	**PPUE (μmol CO_2_ g^−1^ P s^−1^)**
41.2° S	Ec	10.6 (0.3) Aa	6.1 (0.2) ABb	86.2 (2.8) Ab
(Poorest site)	Ga	7.9 (1.0) Aa	5.4 (0.7) ABa	69.5 (7.8) ABa
	Lf	7.7 (0.5) Aa	4.5 (0.3) Ba	58.8 (3.9) Bb
	Lh	8.7 (0.8) Aa	7.4 (0.7) Aa	85.7 (7.5) ABa
41.5° S	Ec	12.8 (0.5) Aa	9.9 (0.8) Aa	168.8 (11.3) Aa
(Richest site)	Ga	8.1 (0.5) BCa	4.5 (1.1) Bb	87.1 (12.2) Ca
	Lf	6.7 (0.3) Cb	5.5 (0.4) Ba	136.4 (10.0) ABa
	Lh	10.2 (0.7) ABa	4.5 (0.9) Bb	102.7 (15.2) BCa

*Each value corresponds to a mean of four samples ± standard error in brackets. Different capital letters indicate significant differences among species within the same site and different lower-case letters indicate significant differences among sites within the same species (P ≤ 0.05)*.

## Discussion

### Leaf nitrogen and phosphorus concentrations and resorption efficiency of SS american proteaceae species

In our study, the semi-deciduous species *Embothrium coccineum* (Supplementary Table S1) reached the highest P and N concentration in the leaves (Supplementary Table S2) and the highest P and N resorption efficiency compared to the other evergreen Proteaceae species evaluated (Figure 2). In contrast, *Orites myrtoidea*, a shrub with perennial and coriaceous leaves, presented the lowest P and N concentration in the leaves (Supplementary Table S2). These results are in agreement with those of several other authors (Reich et al., 1999; Diehl et al., 2008; Gallardo et al., 2012), who reported that species having short leaf lifespan (e.g., broad-leaved deciduous species) have higher leaf P and N concentration due to higher nutrient requirements, and also higher nutrient resorption efficiency. This has been acknowledged as a nutrient conservation strategy, which is less obvious among species with a long leaf lifespan (e.g., broad-leaved evergreen).

Interestingly, the highest P and N resorption efficiency (74.5 and 76.9%, respectively) in *E. coccineum* (Figure 2) was found in the coldest environment of the highest latitude (55.22° S), similar to findings by Oleksyn et al. (2003) in six populations of *Pinus sylvestris* growing in a wide geographic distribution. These authors proposed that plant species inhabiting cold environments increase their internal nutrient cycling because low soil temperatures can limit the mineralization of organic matter and the nutrient release from mineral soils. Indeed, the southernmost site (55.22° S) of our study was one of the sites with the lowest organic carbon content and total N in the soil (Table 2). Thus, our results suggest that the interaction of low temperatures with low soil nutrient availability favor the high nutrient resorption in the leaves of *E. coccineum*, the only SS American Proteaceae species capable of growing in the southernmost, harsh environment (Table 1). Although, this tendency was not clearly observed in other species inhabiting cold environments (37.23° S site), such as *O. myrtoidea* and *L. hirsuta* (Table 1, Figure 2), the results illustrated in the PCA (Figure 1) showed that the temperature is a driver of P resorption efficiency. Among the other edapho-climatic factors examined, those that influenced significantly the P and N resorption were soil pH and Al saturation, variables that have been commonly linked to nutrient availability in the soil (Lambers et al., 2008a)

In general, for all species and sites the P and N concentrations in mature leaves of SS American Proteaceae were on average 0.59 and 11.7 mg g^−1^, respectively. These values are higher compared with P and N concentrations found in leaves of plants from SWA and South Africa (Lambers et al., 2010) and are 1.5 and 2.0 times higher, for N and P respectively, than those found in *Banksia* species (Proteaceae) growing in their natural habitat in SWA (Hayes et al., 2018). Probably this is because SS American Proteaceae species have evolved in nutrient richer soils than Proteaceae species from old, climatically buffered, infertile landscapes such as SWA and South Africa (Hopper, 2009; Lambers et al., 2012a). In this context, we found that, for all species and sites, the P and N resorption efficiencies of SS American Proteaceae were on average 47.7 and 50.6%, respectively, being these values lower compared to those in other Proteaceae species inhabiting ancient and severely P-impoverished soils, such as those in SWA. There, the species exhibit very high nutrient resorption efficiency, especially for P (Lambers et al., 2015a). Some examples of SW Australian Proteaceae species with extremely efficient P resorption are *Banksia chamaephyton* (82%, Denton et al., 2007), *Hakea prostrata* (85%, Shane et al., 2014), *B. attenuata* (90.8%, Hayes et al., 2014), and *B. menziesii* (90.2%, Hayes et al., 2014). These species are frequently found in old, nutrient-impoverished soils such as those along the two-million-year old dune chronosequence in SWA (Hayes et al., 2014).

Studies on long-term soil development and its influence on the vegetation have come to the conclusion that nutrient resorption efficiency in leaves is widely linked to soil age and nutrient availability, and it becomes higher in old and highly weathered soils with low nutrient availability compared to nutrient richer and younger soils (Crews et al., 1995; Richardson et al., 2004; Hayes et al., 2014). However, this apparent negative correlation between nutrient resorption efficiency and nutrient availability in the soil does not always occur. Indeed, our results showed no correlation between nutrient resorption efficiency and soil nutrient availability. Similar results were found by Aerts (1996), who showed that there is no relationship between nutrient resorption efficiency and leaf nutrient status (defined by the author as soil fertility) in several evergreen shrubs and tree species from USA and Europe. Likewise, Gallardo et al. (2012), found that P and N concentrations in leaves and those in the soil are decoupled along a 60,000 years chronosequence in Llaima Volcano, Chile. Probably, in this young chronosequence and in our study sites the soil nutrient availability is not as poor as in ancient landscapes in SWA (Lambers et al., 2006, 2012a) due to rejuvenating catastrophic disturbances occurred in this region (e.g., volcanic eruptions, glaciation, and landslides caused by earthquakes) that have increased the soil nutrient content (Lambers et al., 2008b). Hayes et al. (2014) reported that Proteaceae species are dominant in the poorest and oldest soils along a two-million-year old dune chronosequence in SWA, with values of total soil P and N ranging from 6.6 to 20.3 mg P kg^−1^ and 240 to 288 mg N kg^−1^, respectively. In contrast, our results showed that SS American Proteaceae species grow in soils containing total values of P and N ranging from 63.1 to 951.6 mg P kg^−1^ and 40 to 10,700 mg N kg^−1^, respectively (Table 2). Despite the fact that Proteaceae species can grow in a wide range of soils, the general trend shows that the average values of total soil P and N are much higher in SSA than those in SWA. With this background information, we suggest that SS American Proteaceae have not developed the ability to be as efficient in nutrient resorption in leaves as SW Australian Proteaceae, probably due to the different evolutionary drivers (e.g., soil fertility) to which they have been exposed. The functional divergence between SS American and SW Australian Proteaceae species has been previously suggested by other authors, who have evidenced differences in cluster root functioning (Delgado et al., 2014) and in the accumulation of P and biomass in seeds (Delgado et al., 2015b). The notion of functional divergence, have recently been reinforced by Hayes et al. (2018), who compared the leaf cell-specific nutrient concentrations in Proteaceae species from SWA, Brazil, and Chile. These authors found that only species from extremely P-impoverished habitats preferentially allocated P to photosynthetic mesophyll cells, suggesting it has evolved as an adaptation to their habitat and that it is not a family-wide trait. Likewise, we suggest that the high nutrient resorption efficiency does not follow a general pattern for all species of the Proteaceae family.

Due to the low resorption efficiency of P and N (< 20%) in *L. hirsuta* reported by Diehl et al. (2003) and other unpublished data for *E. coccineum* by Zuñiga-Feest A., Lambers et al. (2012a) proposed SS American Proteaceae species could act as ecosystem engineers in young soils since they provide nutrients through the deposition of leaf litter. However, recently, Fajardo and Piper (2015) claimed to have proof that *E. coccineum* is not an ecosystem engineer, because shows a higher P and N resorption efficiency (41.2 and 39.2%, respectively) compared to neighboring species in Chilean Patagonia (on average, 9.9 and 17.2%, respectively). Based on our results, where all species showed similar resorption efficiency and had higher or similar values of nutrient resorption than co-occurring natives species from the Andean Patagonia forest (as described by Diehl et al., 2003; Fajardo and Piper, 2015), we believe that there is not yet enough evidence to establish with certainty that SS American Proteaceae are ecosystem engineers. In addition, according to the benchmark levels established by Killingbeck (1996) for senescent leaves (concentration values < 0.04% for P and < 0.7% for N), our results showed that most SS American Proteaceae have a nearly complete resorption of P and N. These results demonstrate high resorption proficiency (expressed as the level to which species can reduce nutrient levels in senescent leaves) in SS American Proteaceae, contrasting with the results found by Diehl et al. (2003) in eight native woody species from Patagonian forest. These authors found that several species, including broad-leaved deciduous species, broad-leaved evergreens and conifers, co-occurring with some Proteaceae species in the south of Chile, were proficient in resorbing N but not P. Thus, we suggest that, although SS American Proteaceae species are not as efficient in the use of nutrients as other Proteaceae inhabiting old and severely P-impoverished soils (e.g., from SWA and South Africa), SSA Proteaceae species are proficient in P compared to other SS American species subjected to similar evolutionary pressures. Therefore, they are highly adapted to grow in P poor soils in this part of the world. Additionally, we suggest that these species could be potential ecosystem engineers in young soils however through different mechanisms, for example, by those involving their cluster roots. This could be, as proposed Delgado et al. (2015a), through the increase of nutrient availability at the rhizosphere level that could facilitate the establishment of species without such specialized roots.

### Nitrogen: phosphorus ratios for SS american proteaceae

Based on the range limits for P and N limitation of vegetation proposed by Koerselman and Meuleman (1996), almost all species were limited by P (N:P ratio >16, Figure 3). Similar results have been reported in other studies including *L. hirsuta* and *L. dentata* (Diehl et al., 2003, 2008; Gallardo et al., 2012). In these reports, the authors agreed that in general woody species growing in volcanic soils are limited by N, whereas the Proteaceae species are limited by P. These authors suggest that the cluster roots of this species are not as efficient in P uptake as those of other Proteaceae species growing in old, climatically buffered, infertile landscapes. Thus, they hypothesized that those root structures could be more likely involved in the uptake of N. Indeed, some studies reveal that N deficiency promotes cluster root formation in some Proteaceae species such as *Hakea actities* (W.R. Barker) (Schmidt et al., 2003) and in *E. coccineum* growing on moraine deposits originating from glacial erosion of the Exploradores Glacier, in Chilean Patagonia (Piper et al., 2013). Additionally, some authors reported that organic compounds released by cluster roots into the rhizosphere can become available as substrate for microorganisms (Ryan et al., 2001) and therefore, it could be that these plants are selecting their bacteria to actively benefiting from them. Along these lines, Lamont et al. (2014), further suggested that the formation of cluster roots is stimulated by the presence of plant-growth-promoting rhizobacteria, such as the N-fixing bacteria, which could have a positive effect on the uptake of N and help explain the P limitation we observed in the leaves instead of a limitation of N, as we expected. However, the mechanism by which cluster roots could be taking up N from the soil has not been studied yet in SS American Proteaceae species and further research is needed to understand functioning of cluster roots in these species.

### Photosynthetic nutrient use efficiency per leaf unit, of nitrogen (PNUE) and phosphorus (PPUE)

The nutrient use efficiency found in some plants may be crucial to thrive in soils with nutrient limitations. For instance, SW Australian Proteaceae species are a good example of nutrient use efficient plants because they can successfully subsist with very low P concentrations in their mature leaves but still maintaining a very high PPUE (Denton et al., 2007; Lambers et al., 2010, 2012b, 2015a). In general, our results have revealed lower values of PPUE and PNUE for SS American Proteaceae species (Table 3) than those reported by Lambers et al. (2012a) for SW Australian Proteaceae species, for which PPUE and PNUE values reached 247 ± 30 μmol CO_2_ g^−1^ P s^−1^ and 6.9 ± 0.9 μmol CO_2_ g^−1^ N s^−1^, respectively. These differences between Proteaceae species from SSA and SWA could be explained because they evolved in completely different habitats, with dissimilar climates and edaphic conditions, which in turn could be evidenced by differences in leaf traits. For example, some studies have associated high LMA with high nutrient use efficiency in plants from nutrient-poor habitats by reducing nutrient losses due to the decrease of herbivory (Lambers et al., 2010). In this context, we found that SS American Proteaceae species have lower LMA values (from 141 to 212 g m^−2^, Supplementary Table S1) than plants from SW Australia (from 328 to 498 g m^−2^) and South Africa (from 200 to 276 g m^−2^), which exhibit the highest LMA values in the world (Lambers et al., 2010). These findings support the idea that SW Australian and South African Proteaceae are adapted to thrive in much more nutrient-poor soils than SS American Proteaceae species. In addition, it is interesting to note that the higher PPUE in thick leaves of SW Australian Proteaceae of the genus *Banksia* is associated with the presence of sunken stomata (Lambers et al., 2012b). These leaf structures increase photosynthetic rates by reducing the diffusion pathway of CO_2_ to mesophyll cells (Hassiotou et al., 2009; Lambers et al., 2015c). Sunken stomata are absent in species with thinner leaves such as *E. coccineum, G. avellana, L. ferruginea, L. hirsuta* (Hayes et al., 2018), which could explain, at least in part, the lower nutrient use efficiency in SS American Proteaceae species. High leaf lifespan values has also been associated with high nutrient use efficiency in plants from nutrient-poor soils by maintaining the nutrients for a longer period (Denton et al., 2007; Lambers et al., 2010). However, this assumption is not so clear for Proteaceae because SS American species (except *E. coccineum*) exhibit similar or higher leaf lifespan (Supplementary Table S1) than the SW Australian Proteaceae species described (Denton et al., 2007). This knowledge suggest that the strategy to use the nutrients efficiently of SS American Proteaceae species is through the maintenance of the nutrients in their leaves for longer period of time (especially *G. avellana*, Supplementary Table S1).

Contrary to our expectations, we found that *E. coccineum* and *L. ferruginea* have higher PPUE and PNUE in the nutrient richest site than in the nutrient poorest one. This probably occurs because in the nutrient richest site these species have significantly high SLA compared to the nutrient poorest site (Table 4). In concordance, Wright et al. (2004) stated that at a global scale, species with thinner leaves have shorter diffusion paths from stomata to chloroplasts favoring the photosynthesis process on an leaf area basis. Likewise, Poorter and Evans (1998) reported that several plant species (as trees, shrubs, and herbs) with high SLA have higher PNUE than that of low SLA species, mainly due to: lower N content per unit of leaf area, larger allocation of organic N to thylakoids and rubisco, and higher rubisco specific activity. Among our studied species, *E. coccineum* had the highest SLA in almost all studied sites (Table 4), and showed a negative correlation to N content per leaf area (*R*^2^ = −0.4942), suggesting that the high PNUE present in *E. coccineum* could follow a similar physiological pattern to those species with high SLA reported by Poorter and Evans (1998). It should be noted that although *G. avellana* and *L. hirsuta* maintained their SLA values at both contrasting sites (41.2° S and 41.5° S, Table 4) their PNUE was higher in the poorest one (Table 3). These results showed that N use efficiency increases in the leaves of *G. avellana* and *L. hirsuta* when nutrient availability in the soil decreases, which has also been observed in other forest tree species (Boerner, 1984). This relationship was not observed in the PPUE neither in the other species studied, suggesting that a high nutrient use efficiency in the poorest soils is not a general response among all SS American Proteaceae species.

**Table 4:** Specific leaf area (SLA) and P and N concentration per unit of leaf area of *Embothrium coccineum* (Ec), *Gevuina avellana* (Ga), *Lomatia ferruginea* (Lf), *Lomatia dentata* (Ld), *Lomatia hirsuta* (Lh), and *Orites myrtoidea* (Om) growing in their natural habitat.

**Siteanish (S Latitude)**	**Species**	**SLAanish (cm^2^ g^−1^)**	**[P] per leaf areaanish (μg cm^−2^)**	**[N] per leaf areaanish (μg cm^−2^)**
37.2° S	Om	50.1 (3.4) A-	9.9 (0.0) A-	170.0 (23.8) A-
	Lh	51.9 (1.3) Aa	9.6 (0.0) Aa	122.9 (11.5) Aa
37.4° S	Ec	79.6 (6.8) Aab	8.5 (0.4) Abc	202.3 (7.9) Aabc
	Ga	59.5 (1.3) Ba	7.0 (0.5) Aa	172.5 (12.9) Bb
	Lf	66.8 (3.3) ABbc	6.5 (0.3) Ab	142.9 (8.7) Bc
	Lh	59.8 (3.2) Ba	7.8 (1.1) Aa	168.7 (3.8) ABa
	Ld	71.3 (5.0) AB-	6.7 (0.8) A-	139.0 (4.3) AB-
40.3° S	Ec	133.4 (7.7) Aa	2.7 (0.4) Ac	134.1 (9.9) ABbc
	Ga	65.6 (1.6) Ba	7.2 (0.9) Aa	157.0 (21.3) Ab
	Lf	130.5 (2.7) Aa	3.7 (0.3) Ab	91.1 (5.4) Bc
41.1° S	Ec	63.2 (2.7) Acd	12.7 (1.7) Aa	177.6 (16.3) Aabc
	Ga	56.0 (1.0) ABa	10.4 (2.8) Aa	146.2 (15.6) Ab
	Lf	40.5 (0.8) Cc	13.1 (3.0) Aa	170.1 (25.2) Abc
	Lh	51.1 (3.5) BCa	12.4 (1.2) Aa	142.2 (20.6) Aa
41.5° S	Ec	88.4 (8.1) Ab	7.6 (1.0) ABbc	117.0 (7.7) Aa
	Ga	65.8 (5.3) Ba	9.0 (1.0) ABa	183.9 (1.1) Aab
	Lf	94.2 (9.1) Aab	4.9 (1.0) Bb	120.5 (2.7) Abc
	Lh	63.3 (4.5) Ba	9.9 (0.9) Aa	193.6 (46.1) Aa
42.1° S	Ec	72.6 (4.6) Abcd	11.1 (1.6) Aab	237.1 (18.3) Aab
	Ga	53.7 (2.6) ABa	12.5 (3.8) Aa	219.8 (16.1) Aa
	Lf	47.7 (2.0) Bbc	8.2 (0.3) Aab	221.6 (17.6) Aab
42.4° S	Ec	75.4 (1.6) Abcd	10.1 (2.17) Aabc	250.1 (13.6) Aab
	Lf	48.4 (2.5) Abc	11.6 (0.38) Aab	276.4 (13.2) Aa
45.3° S	Ec	69.4 (2.1) Abcd	14.4 (2.6) Aab	200.3 (4.2) Aabc
	Lf	89.3 (14.1) Aab	9.7 (1.6) Aab	155.7 (27.5) Abc
51.2° S	Ec	59.7 (2.2) d	15.0 (0.5) a	253.5 (20.2) a

*Each value corresponds to a mean of 4–10 samples ± standard error in brackets. Different capital letters indicate significant differences among species within the same site and different lower-case letters indicate significant differences among sites within the same species (P ≤ 0.05)*.

## Conclusion

Our study showed a unique data set related to nutrient (P and N) resorption and photosynthetic nutrient (P and N) use efficiency in the six SS American Proteaceae species inhabiting across a large geographical range. First, we have showed that P and N resorption efficiency in leaves of Proteaceae species is not correlated to the P and N content of the soil. Second, SS American Proteaceae species are more limited by P than N as suggested by the N:P ratio in leaves. Third, PPUE and PNUE showed variations among the SS American Proteaceae evaluated, being *E. coccineum* the species presenting the highest values. Nevertheless, and contrary to our expectations, the PPUE and PNUE were, in general, higher in the nutrient richest soil. All these findings will help us to better understand the functioning of SS American Proteaceae species, which did not show a general pattern in the nutrient use efficiency among them neither with others Proteaceae species reported in the literature.

## Author contributions

MD, this author contributed to the conception of this study, organized and carried out the field trips to collect the data. Besides, she organized (i.e., Graphs, tables) and interpreted the data along with writing the manuscript at all stages until the final version, given approval to be submitted. SV, this author contributed to the field data collection, statistical analysis, valuables comments and revisions on the manuscript at all stages. MR, this author contributed with funding to do some chemical analysis in the leaf samples and revised critically the manuscript at the final stage. PB, this author contributed to statistical analysis, and helped in the design of figures, and writing of the manuscript. AZ-F, this author participated in the conception of this study. Besides contributed to the field data collection, funding to perform chemical analysis of soil and leaf samples and contributed with important intellectual content at all stages. All authors revised the manuscript and approved the final version.

### Conflict of interest statement

The authors declare that the research was conducted in the absence of any commercial or financial relationships that could be construed as a potential conflict of interest.
