# Supplementary material for: Nutrient Use Efficiency of Southern South America Proteaceae Species. Are there General Patterns in the Proteaceae Family?
Source: Front Plant Sci. 2018 Jun 27;9:883. doi: 10.3389/fpls.2018.00883 (PMC6030812; doi:10.3389/fpls.2018.00883)
Supplement: Supplementary file 1 [file Table_1.docx]

**Supplementary data for “Nutrient Use Efficiency of Southern South America Proteaceae Species. Are there General Patterns in the Proteaceae Family?”**

**Authors:** Delgado, M., Valle, S., Reyes-Díaz, M., Barra P.J., Zúñiga-Feest, A.

**Supplementary Table S1.** Characteristics of foliage of *Embothrium coccineum* (Ec), *Gevuina avellana* (Ga), *Lomatia ferruginea* (Lf), *Lomatia dentata* (Ld), *Lomatia hirsuta* (Lh), and *Orites myrtoidea* (Om).

| Species | Shade tolerance level* | Type of foliage* | type of leaf | Mean leaf area  (cm^2^) | Leaf mass per unit area (g m^-2^) | Mean leaf lifespan (years)** |
| --- | --- | --- | --- | --- | --- | --- |
| *Ec* | Intolerant | Semi- deciduous | Simple | 18.0 (1.07) | 140.9 (3.8) | 0.7 - 0.9 |
| *Lf* | Tolerant | Evergreen | Compound | 207.6 (30.9) | 177.5 (7.9) | 2.4 - 3.4 |
| *Ga* | Mid-tolerant | Evergreen | Compound | 290.5 (38.8) | 177.2 (5.4) | 4.3 - 5.4 |
| *Ld* | Tolerant | Evergreen | Simple | 10.0 (1.1) | 145.4 (8.5) | n.d. |
| *Lh* | Intolerant | Evergreen | Simple | 38.0 (2.4) | 177.4 (5.1) | 2.3 |
| *Om* | Intolerant | Evergreen | Simple | 2.0 (0.1) | 212.0 (17.0) | n.d. |

* Data from Donoso, C. (2006). Las especies arbóreas de los bosques templados de Chile y Argentina, Autoecología. Valdivia, Chile.

**Data from Lusk, C.H., and Corcuera, L.J. (2011). Effects of light availability and growth rate on leaf lifespan of four temperate rainforest Proteaceae. *Rev. Chil. Hist. Nat*. 84, 269-277.

n.d. = not determined
