# Supplementary material for: Nutrient Use Efficiency of Southern South America Proteaceae Species. Are there General Patterns in the Proteaceae Family?
Source: Front Plant Sci. 2018 Jun 27;9:883. doi: 10.3389/fpls.2018.00883 (PMC6030812; doi:10.3389/fpls.2018.00883)
Supplement: Supplementary file 2 [file Table_2.docx]

**Supplementary data for “Nutrient Use Efficiency of Southern South America Proteaceae Species. Are there General Patterns in the Proteaceae Family?”**

**Authors:** Delgado, M., Valle, S., Reyes-Díaz, M., Barra P.J., Zúñiga-Feest, A.

**Supplementary Table S2.** Phosphorus (P) and nitrogen (N) concentrations per gram of dry weight (DW) in mature and senescent leaves of *Embothrium coccineum* (Ec), *Gevuina avellana* (Ga), *Lomatia ferruginea* (Lf), *Lomatia dentata* (Ld), *Lomatia hirsuta* (Lh), and *Orites myrtoidea* (Om) growing in their natural habitat*.* Each value corresponds to a mean of four to ten samples ± standard error in brackets. Different capital letters indicate significant differences among species within the same site and different lower-case letters indicate significant differences among sites within the same species (*P*≤0.05).

| **Site**  **(S Latitude)** | **Species** | **Mature** | | **Senescent** | |
| --- | --- | --- | --- | --- | --- |
|  |  | **N**  **(mg g^-1^ DW )** | **P**  **(mg g^-1^ DW)** | **N**  **(mg g^-1^ DW)** | **P**  **(mg g^-1^ DW)** |
| 37.2º S | Om | 7.11 (1.12) A- | 0.37 (0.02) A- | 3.05 (0.49) A- | 0.22 (0.08) A- |
|  | Lh | 6.38 (0.60) Ab | 0.35 (0.02) A- | 3.61 (0.70) Abc | 0.21 (0.01) A- |
|  |  |  |  |  |  |
| 37.4º S | Ec | 16.10 (0.63) Aab | 0.76 (0.03) Aa | 7.20 (2.17) Abc | 0.28 (0.02) Aabc |
|  | Ga | 10.26 (0.77) Ba | 0.36 (0.02) Ba | 6.39 (0.30) Aa | 0.27 (0.02) Aa |
|  | Lf | 9.55 (0.58) Bbc | 0.40 (0.01) Ba | 5.84 (0.32) Aa | 0.20 (0.01) Aa |
|  | Ld | 9.96 (0.31) B- | 0.35 (0.01) B- | 6.87 (0.33) A- | 0.31 (0.00) A- |
|  | Lh | 10.26 (0.32) Bb | 0.46 (0.06) Ba | 6.60 (0.20) Aa | 0.24 (0.03) Aa |
|  |  |  |  |  |  |
| 40.3º S | Ec | 17.90 (1.30) Aab | 0.56 (0.05) Aa | 6.30 (0.60) Aabcd | 0.18 (0.01) Aabc |
|  | Ga | 12.00 (1.40) Aa | 0.41 (0.05) Aa | 6.00 (1.10) Aa | 0.23 (0.03) Aa |
|  | Lf | 11.80 (0.50) Aab | 0.44 (0.04) Aa | 4.60 (0.40) Aab | 0.27 (0.01) Aa |
|  |  |  |  |  |  |
| 41.1º S | Ec | 12.49 (1.76) Ab | 0.89 (0.14) Aa | 4.34 (0.74) Ad | 0.43 (0.11) Aa |
|  | Ga | 8.19 (0.87) Aa | 0.58 (0.16) Aa | 4.02 (0.36) Aa | 0.38 (0.17) Aa |
|  | Lf | 6.89 (1.02) Bc | 0.53 (0.12) Aa | 3.82 (0.35) Ab | 0.20 (0.03) Aa |
|  | Lh | 5.99 (1.52) ABb | 0.54 (0.11) Aa | 2.14 (0.54) Ac | 0.14 (0.01) Aa |
|  |  |  |  |  |  |
| 41.5º S | Ec | 10.40 (0.80) Ab | 0.67 (0.09) Aa | 5.20 (0.20) Acd | 0.22 (0.02) Abc |
|  | Ga | 12.10 (0.10) Aa | 0.59 (0.06) Aa | 5.40 (0.90) Aa | 0.26 (0.04) Aa |
|  | Lf | 11.40 (0.30) Aab | 0.46 (0.10) Aa | 4.50 (0.20) Aab | 0.31 (0.07) Aa |
|  | Lh | 12.30 (2.90) Aa | 0.63 (0.06) Aa | 4.60 (0.30) Ab | 0.17 (0.04) Aa |
|  |  |  |  |  |  |
| 42.1º S | Ec | 17.20 (1.30) Aab | 0.80 (0.11) Aa | 7.20 (0.20) Abc | 0.32 (0.04) Aabc |
|  | Ga | 11.80 (0.90) Ba | 0.67 (0.20) ABa | 6.70 (0.30) Aa | 0.51 (0.18) Aa |
|  | Lf | 10.60 (0.80) Bab | 0.39 (0.01) Ba | 6.20 (0.40) Aa | 0.26 (0.02) Aa |
|  |  |  |  |  |  |
| 42.4º S | Ec | 18.85 (1.03) Aa | 0.76 (0.16) Aa | 10.11 (0.47) Aa | 0.39 (0.06) Aab |
|  | Lf | 13.38 (0.64) Ba | 0.53 (0.03) A- | 6.45 (0.41) Ba | 0.29 (0.02) Aa |
|  |  |  |  |  |  |
| 45.3º S | Ec | 13.90 (0.30) Aab | 0.99 (0.15) Aa | 9.54 (1.80) Aab | 0.56 (0.02) Aa |
|  | Lf | 12.80 (0.30) Aab | 0.88 (0.32) Aa | 5.90 (0.50) Aab | 0.33 (0.08) Ba |
|  |  |  |  |  |  |
| 51.2º S | Ec | 13.87 (1.58) -ab | 0.88 (0.03) -a | 3.70 (0.40) -d | 0.27 (0.05) -b |
